# Cytosolic group IVA phospholipase A2 inhibitors, AVX001 and AVX002, ameliorate collagen-induced arthritis

**DOI:** 10.1186/s13075-018-1794-6

**Published:** 2019-01-21

**Authors:** A. J. Feuerherm, E. A. Dennis, B. Johansen

**Affiliations:** 1 Department of Biology, Norwegian University of Science and Technology, N-7491 Trondheim, Norway; 2 Department of Chemistry and Biochemistry, University of California−San Diego, La Jolla, California, 92093-0601 USA; 3 Department of Pharmacology, School of Medicine, University of California−San Diego, La Jolla, California, 92093-0601 USA

**Keywords:** cPLA2α, Disease-modifying, Anti-rheumatic, Small molecule inhibitors

## Abstract

**Background:**

Cytosolic phospholipase A2 group IVA (cPLA2α)-deficient mice are resistant to collagen-induced arthritis, suggesting that cPLA2α is an important therapeutic target. Here, the anti-inflammatory effects of the AVX001 and AVX002 cPLA2α inhibitors were investigated.

**Methods:**

In vitro enzyme activity was assessed by a modified Dole assay. Effects on inhibiting IL-1β-induced release of arachidonic acid (AA) and prostaglandin E2 (PGE2) were measured using SW982 synoviocyte cells. In vivo effects were studied in prophylactic and therapetic murine collagen-induced arthritis models and compared to methotrexate (MTX) and Enbrel, commonly used anti-rheumatic drugs. The in vivo response to treatment was evaluated in terms of the arthritis index (AI), histopathology scores and by plasma levels of PGE2 following 14 and 21 days of treatment.

**Results:**

Both cPLA2α inhibitors are potent inhibitors of cPLA2α in vitro. In synoviocytes, AVX001 and AVX002 reduce, but do not block, release of AA or PGE2 synthesis. In both CIA models, the AI and progression of arthritis were significantly lower in the mice treated with AVX001, AVX002, Enbrel and MTX than in non- treated mice. Several histopathology parameters of joint damage were found to be significantly reduced by AVX001 and AVX002 in both prophylactic and therapeutic study modes; namely articular cavity and peripheral tissue inflammatory cell infiltration; capillary and synovial hyperplasia; articular cartilage surface damage; and periostal and endochondral ossification. In comparison, MTX did not significantly improve any histopathology parameters and Enbrel only improved ossification. Finally, as a biomarker of inflammation and as an indication that AVX001 and AVX002 blocked the cPLA2α target, we determined that plasma levels of PGE2 were significantly reduced in response to the AVX inhibitors and MTX, but not Enbrel.

**Conclusions:**

AVX001 and AVX002 display potent anti-inflammatory activity and disease-modifying properties in cellular and in vivo models. The in vivo effects of AVX001 and AVX002 were comparable to, or superior, to those of MTX and Enbrel. Taken together, this study suggests that cPLA2α inhibitors AVX001 and AVX002 are promising small molecule disease-modifying anti-rheumatic therapies.

## Introduction

Rheumatoid arthritis (RA) is an autoimmune and systemic inflammatory disease affecting 0.5–1% of the population worldwide. RA primarily affects the synovial joints, but extra-articular manifestations are also common. The disease is initiated in the synovium of synovial joints and progresses into chronic synovitis that damages the articular cartilage and underlying bone, leading to pain and loss of joint function [1, 2]. RA aetiology is multifactorial with an unknown triggering agent, and proinflammatory cytokines and chemokines are recognized to play critical roles in the pathogenesis [3]. Excessive levels of pro-inflammatory cytokines including tumor necrosis factor (TNF) and interleukin 1β (IL-1β) are found in the joint [2, 4]. In the synovium in RA, TNF and IL-1β elicit a variety of biological effects on inflammation and joint destruction [4]. TNF has been found to regulate the expression and pathophysiological function of many factors that contribute to inflammation and joint destruction; e.g. the interleukin IL-6, the chemokines IL-8 and CCL2/MCP-1 [5, 6] and the matrix metalloproteinases MMP1 and MMP3 [7].

We have previously shown that TNF and IL-1β activate phospholipase A2 (PLA2) enzymes and that this event regulates important aspects of inflammation in several cell types [8–13]. PLA2 enzymes constitute a superfamily of lipolytic enzymes that catalyze the hydrolysis of membrane phospholipids yielding lysophospholipids and free fatty acids [14]. Group IVA cPLA2 (cPLA2α) is selective for arachidonic acid (AA) in the phospholipid *sn*-2 position, and is essential in agonist-induced AA release as the major contributor to increased levels of free AA in inflammation [15–17]. Following cPLA2α activation, free AA is enzymatically metabolized into bioactive eicosanoids, of which prostaglandin E2 (PGE2) is a central lipid mediator in arthritis [18–21].

Non-steroidal anti-inflammatory drugs (NSAIDs) are widely used to relieve rheumatic symptoms, however, with several serious adverse effects such as increased risk of gastrointestinal events, myocardial infarction and cerebrovascular events [22–26]. The NSAID mechanism of action was first described in 1971 by Vane and Piper who demonstrated that NSAIDs exert their effects through inhibition of prostaglandin and prostanoid biosynthesis by cyclooxygenase (COX) enzymes. NSAIDs alleviate pain and reduce fever by inhibiting prostaglandin (PG) G/H synthases, commonly known as COXs. The analgesic efficacy of NSAIDs is largely attributable to reduced levels of COX2-derived PGE2 and PGI2 [27, 28]. According to recent recommendations, prescription of NSAIDs in rheumatic patients should be done with caution to reduce the risk of cardiovascular disease (CVD) events [29]. Rather, the most recent recommendations states that treatment with DMARDS should be initiated as soon as RA is diagnosed [30]. Of the conventional synthetic disease-modifying antirheumatic drugs (csDMARDs), methotrexate (MTX) is considered as the anchor drug to treat RA [31], either as monotherapy or in combination with NSAIDs or biological or targeted synthetic DMARDS [32, 33]. To date, biologics targeting TNF (infliximab/Remicade™, etanercept/Enbrel™, adalimumab/Humira™, golimumab/Simponi™, certolizumab/Cimzia™) are the most efficient anti-rheumatic drugs, however approximately one third of patients do not respond successfully to treatment [34] or become resistant [35]. Anti-TNF therapies are also monitored closely due to reports of malignancies, serious infections and long-term safety concerns [3, 36], emphasizing the need for novel therapeutic options.

As the rate-limiting factor for AA release, positioned upstream of the AA-metabolizing enzymes, cPLA2α has for long been considered as an attractive therapeutic target in chronic diseases including RA [17, 37–40]. In 1993, arachidonyl trifluoromethyl ketone (ATK, AACOCF3) was presented as the first synthetic inhibitor of cPLA2α [41]. Since then, several cPLA2α specific inhibitors have been synthesized and characterized (reviewed in [42–45]) and numerous in vivo studies employing different tools and agents to inhibit cPLA2α activity [37–40] support that cPLA2α is indeed an attractive therapeutic target to alleviate pain and disability and thereby also reduce the social and economic burden that this disease places on the individual and on society [46].

Given the limitations with today’s anti-rheumatic treatment regimes [30, 47–49], the potential use of cPLA2α specific inhibitors as alternative or adjunct anti-inflammatory therapeutics is of great interest. We have recently described in vitro and in vivo anti-inflammatory and anti-angiogenic effects of the oxothiazol compound Inhibitor 28 [50, 51]. We have also previously reported in vitro inhibitory and anti-inflammatory cellular effects of the docosahexaenoic acid derivatives AVX001 and AVX002 [11–13]. In this study, our objective was to further examine the effects of AVX001 and AVX002 in vitro, in a cellular model of synovitis, and in vivo in the collagen-induced arthritis (CIA) mouse model [52–54], and to compare their effects with those of the commonly used anti-rheumatic therapapies Enbrel and MTX [32, 33].

## Materials and methods

### Compounds

Recombinant IL-1β was obtained from Roche (#11457756001). AVX001 ((6E,10Z,13Z,16Z,19Z)-1,1,1-trifluoro-4-thiadocosa-6,10,13,16,19-pentaen-2-one) and AVX002 (1,1,1-trifluoro-3-(((3Z,6Z,9Z,12Z,15Z)-octadeca-3,6,9,12,15-pentaen-1-yl)thio)propan-2-one) were synthesized by Synthetica AS, Oslo [55]. Docosahexaenic acid was from Cayman Chemicals (#90310), MTX from Sigma Aldrich (#A6760), Enbrel/Etanercept from Boehringer Ingelheim Pharma KG (#F39487), and dimethyl sulfoxide (DMSO) from Sigma Aldrich (#D2650).

### In vitro mixed micelle assay

The activity of cPLA2α (group IVA), iPLA2 (group VIA) and sPLA2 (group V) was determined using a modified Dole assay [56–58]. Buffers and substrates were optimized for each enzyme assay as follows: (a) GIVA cPLA2: 400 μM Triton X-100, 97 μM 1-palmitoyl-2-arachidonoyl-sn-glycero-3-phosphocholine (PAPC), 1.8 μM ^14^C-labeled PAPC, and 3 μM phosphatidylinositol-4,5-bisphosphate (PIP_2_) in 100 mM HEPES buffer, pH 7.5, with 90 μM CaCl_2_, 2 mM dithiothreitol (DTT), and 0.1 mg/mL bovine serum albumin (BSA); (b) GVIA iPLA2: 400 μM Triton X100, 98.3 μM PAPC, and 1.7 μM ^14^C-labeled PAPC in buffer containing 100 mM HEPES, pH 7.5, 2 mM ATP, and 4 mM DTT; and (c) GV sPLA2: 400 μM Triton X-100, 98.3 μM PAPC, and 1.7 μM ^14^C-labeled PAPC in buffer containing 50 mM Tris, pH 8.0, and 5 mM 550 CaCl_2_.

### Cell culture

The human synovial sarcoma-derived cell line SW982 (ATCC, London, UK) was maintained as described previously [12]. All experiments were performed at 3 days post confluency in serum-free medium following overnight serum starvation to ensure synchronization of the cells. For arachidonic acid and oleic acid release, serum starvation included the addition of [^3^H]-arachidonic acid and [^14^C]- oleic acid (NEN Perkin Elmer); see [12] for details on the release assay. Cells were pre-treated for 2 h AVX001 and AVX002 before the addition of IL-1β (10 ng/mL). For PGE2 analysis, cell supernatants were aspired after 24 h of IL-1β stimulation, centrifuged to remove cell debris, aliquoted and stored at − 80 °C until analysis.

### PGE2 analysis

PGE2 enzyme immunoassay (EIA) analysis of blood plasma from the prophylactic and therapeutic CIA studies and from cell supernatants was performed according to the kit protocol (Cayman, #514010). Plasma samples for PGE2 analysis were diluted 1:1000–1:6000 and cell supernatants were diluted 1:50–1:8000 in EIA buffer and allowed to hybridize overnight (18 h, 4 °C). A Multiscan plate reader (Ascent Labsystems) (optical density (OD) 414 nm) and the corresponding Ascent software for Multiscan, Version 2.4.1 were used to obtain the data before analyzing the data using a 4-parameter logistic fit model. PGE2 mean ± SD values for all treatments are shown relative to the DMSO-treated arthritic mice (*n* = 10–11 mice in each category) or relative to untreated cells (*n* = 2–4 experiments performed in duplicates).

### In vivo studies of cPLA2α inhibitors AVX001 and AVX002

The in vivo studies were conducted in accordance with standard operating procedures based on current International Conference on Harmonization (ICH) Harmonized Tripartite Guidelines [59] and generally accepted procedures for the testing of pharmaceutical compounds. Separate prophylactic and therapeutic efficacy studies of AVX001 and AVX002 were performed. MTX, Enbrel, and vehicle (DMSO 100%) were administered to all groups via intraperitoneal injection once daily at a dose volume of 2 mL/kg body weight. Clinical observations were conducted daily during the study. Biopsies for mid-term histology analysis were obtained on day 13 in the prophylactic study. Necropsy examinations were performed at study termination in both the prophylactic and therapeutic study modes; plasma samples were collected and hind paws were collected for histopathology analyses.

### Induction of CIA

For the prophylactic and the therapeutic studies, CIA was induced in male DBA/1 mice (except in the untreated, naïve mice) by immunization at the tail base with 0.1 mL emulsion containing an equal volume of bovine type II collagen solution (2 mg/mL) and Freuds Complete Adjuvant. The rationale for selecting the doses of 10 mg/kg and 30 mg/kg body weight was based on the reported anti-inflammatory effects with the inhibitor ATK (AACOCF3) in mice, a cPLA2α inhibitor having structural similarities to AVX001 and AVX002 [11, 41, 60]. The first injection was given on day 0 and the second booster injection was given on day 21 [52–54]. Vehicle (DMSO) and MTX (0.3 mg/kg) were administered daily, Enbrel (25 mg/kg) was administrated twice a week, whereas AVX001 and AVX002 were administered daily for the first 4 days of treatment and then every second day until study termination. For the prophylactic study, treatment started 1 h before the second collagen injection and continued for 21 days except in the histology groups that were sacrificed at day 13 (33 days after immunization). Treatment started at day 28 in the therapeutic study and continued for 14 days.

### CIA assessment and treatment

Arthritis was assessed by capacity measurement of paw swelling by two observers blinded to treatment. The occurrence of arthritis was observed by scoring all paws for severity of erythema and swelling, using a clinical score ranging from 0 (no swelling) to 4 (severe swelling and erythema), i.e. yielding a maximum arthritis index (AI) score of 16 [61].

### Measurement of the histopathology and clinical observations

At the end of the studies, the animals were disposed of using carbon dioxide and subjected to necropsy, supervised by a pathologist. Mice were disposed of approximately 5 h after the last injection. A macroscopic examination of all dead animals was performed and any abnormality was recorded. One hind foot from each mouse in each group was collected for histopathology. The foot including the ankle was fixed in 10% neutral formalin. The ankle joints were decalcified, dehydrated, embedded in paraffin, sectioned and stained with routine hematoxylin-eosin. Arthritis damage (histological damage score) was evaluated by light microscopy and scored by an investigator blinded to the treatment regimen. The following histopathology parameters were evaluated: (1) articular cavity and peripheral tissue inflammatory cell infiltration; (2) capillary and synovial hyperplasia; (3) articular cartilage surface damage and (4) endochondral and periostal ossification, each using a 0–5 grading system (0 = none; 1 = minimal; 2 = mild; 3 = moderate; 4 = marked; 5 = severe damage).

### Statistical analysis

Data from each group were examined by one-way analysis of variance, and individual groups were then compared using Student’s unpaired *t* test for normally distributed data. Data were analyzed as mean ± SD; *p* < 0.05 was considered significant.

## Results

### AVX001 and AVX002 are potent and selective inhibitors of cPLA2α

AVX001 and AVX002 are potent inhibitors of cPLA2α activity both in an in vitro vesicular assay and in cellular systems [8–13]. Here, we have directly compared the effects of these inhibitors to their precursor docosahexaenoic acid (DHA) [55] in an in vitro mixed micelle assay to investigate PLA2 inhibition and specificity. The inhibition results are presented in Table 1, either as percent inhibition or as Χ_I_(50) values. At first, the percent of inhibition for each PLA2 enzyme at a 0.091 mol fraction of each inhibitor was determined. Then, the Χ_I_(50) values were determined for compounds that displayed greater than 90% inhibition of GIVA cPLA2. The Χ_I_(50) is the mole fraction of the inhibitor in the total substrate interface required to inhibit the enzyme activity by 50%. As previously shown by a vesicular in vitro assay [11], AVX001 and AVX002 are equally potent in inhibiting cPLA2α activity. In the current study, this finding was confirmed by the mixed micelle in vitro assay, in which a 0.091 mol fraction of the inhibitors yielded at least 90% inhibition, corresponding to the Χ_I_(50) values 0.0072 for AVX001 and 0.0052 for AVX002. Furthermore, we found that the 0.091 mol fraction of the inhibitors did not inhibit iPLA2 or sPLA2 activity as far less than 90% inhibition was reached, hence Χ_I_(50) values were not determined. In contrast to AVX001 and AVX002, the synthetic precursor for AVX001 and AVX002, namely DHA, did not inhibit cPLA2α, iPLA2 or sPLA2 activity.

**Table 1:** In vitro activity of AVX001, AVX002, and DHA in the mixed micelle cPLA2α enzyme assay

Name	Structure	In vitro enzyme activity				
		GIVA cPLA_2_		GVIA iPLA_2_	GV sPLA_2_	GIVA cPLA_2_ vesicle assay
		% Inhibit^a^	XI(50)	% Inhibit^a^	% Inhibit^a^	IC50 (nM)^b^
AVX001		90	0.0072 ± 0.0007	68	62	120 ± 58
AVX002		93	0.0057 ± 0.001	88	68	126 ± 37
DHA		8.9	ND	38	32	ND

*DHA* Docosahexaenic acid, *cPLA* cytosolic phospholipase A2 protein, *IC50* half maximal inhibitory concentration, *ND* not determined

^a^Mixed micelle enzyme in vitro assay: % inhibition at a 0.091 mol fraction of each inhibitor

^b^Vesicle enzyme in vitro data included for reference. Inhibitors were tested in the 0–3 μM range [11]

### AVX001 and AVX002 show long-term inhibition of arachidonic acid and PGE2 release in synoviocytes

**Fig. 1:**
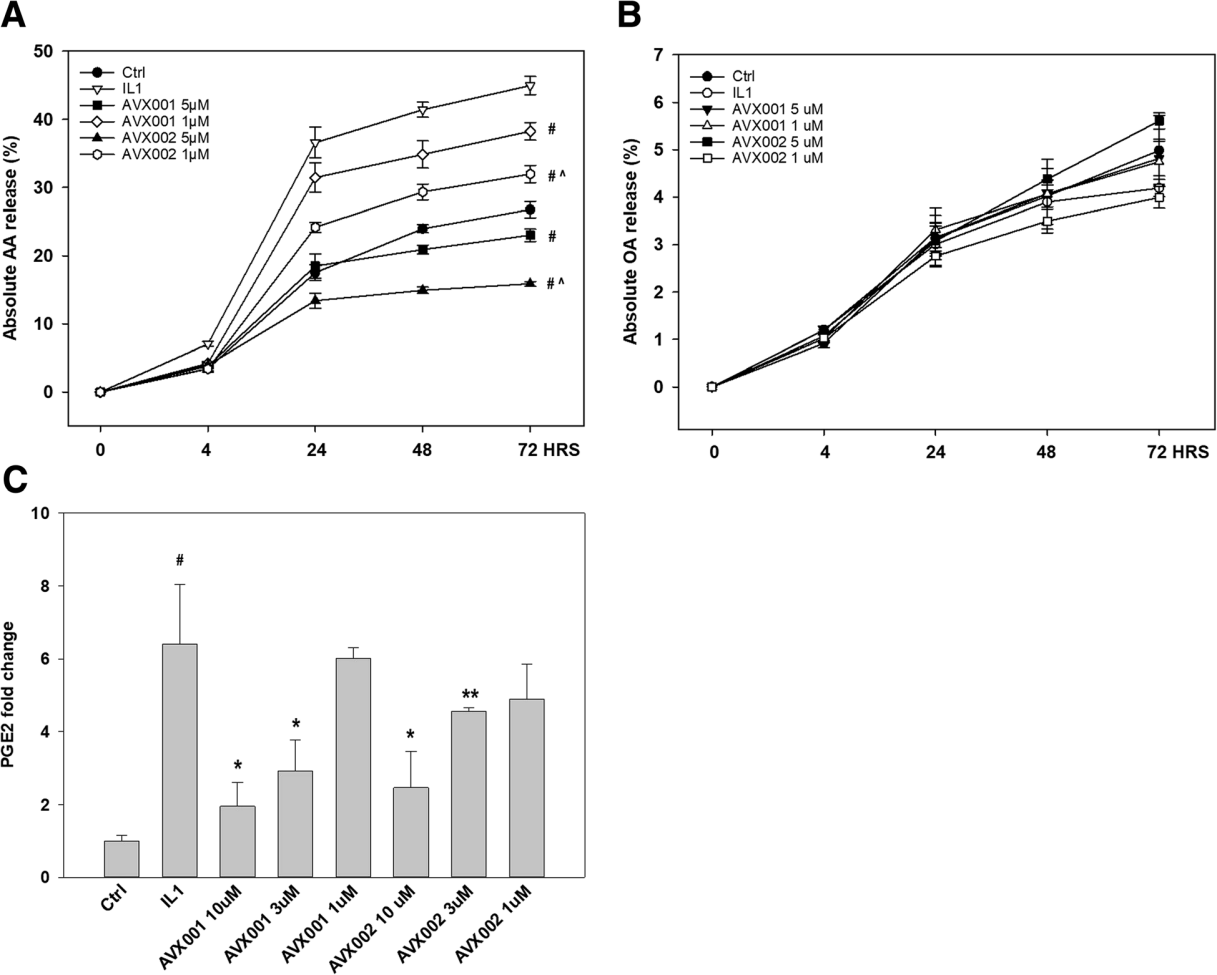
Long-term inhibition of arachidonic acid and prostaglandin E2 (PGE2) release in synoviocytes. **a** Inhibition of arachidonic acid (AA) release by AVX001 and AVX002 in synoviocytes in response to IL-1β (10 ng/mL). ^#^*p* ≤ 0.001 vs. IL-1β, ^^^*p* ≤ 0.001 vs. AVX001. **b** IL-1β, AVX001 and AVX002 do not affect oleic acid (OA) release. **c** AVX001 and AVX002 reduce PGE2 synthesis following 24 h stimulation with IL-1β (10 ng/mL). ^#^*p* ≤ 0.001 vs. control (Ctrl); *p ≤ 0.05 vs. IL-1β; **p ≤ 0.001 vs. IL-1β. Results shown are average ± SD of 2–4 experiments analyzed in duplicates. HRS, hours

AVX001 and AVX002 are potent inhibitors of synoviocyte arachidonic acid (AA) release in response to TNF and toll-like receptor 2 (TLR2) ligands [12, 13]. In time-course studies, AVX001 and AVX002 inhibited IL-1β-induced AA release in a dose-dependent manner with estimated half maximal inhibitory concentration (IC50) values of 1.1 μM and 0.71 μM for AVX001 and AVX002 at 24 h, respectively (4-parameter logistic curves not shown). The inhibitory effect on AA release reached a plateau after 24 h and this effect persisted until the experiments were terminated at 72 h (Fig. 1a). When comparing AVX001 and AVX002 inhibition of AA release, AVX002 was significantly more potent than AVX001. In contrast, oleic acid (OA) release was not affected by IL-1β or by AVX001 or AVX002; no significant induction or inhibition of OA was observed (Fig. 1b), implying that non-arachidonyl selective PLA2 isotypes are not involved or affected. Furthermore, the reduction in AA was in turn also reflected in reduced PGE2 levels (Fig. 1c).

### Prophylactic treatment with AVX001 and AVX002 efficiently delays and reduces arthritis progression

Synthetically modified polyunsaturated fatty acid derivatives such as AVX001 and AVX002 are shown to display potent anti-inflammatory effects in cellular model systems [11, 12, 55, 62]. One objective was to explore the in vivo prophylactic anti-inflammatory effects of cPLA2α inhibitors AVX001 and AVX002 on the CIA model in male DBA/1 mice, a common autoimmune model of RA in which cPLA2α activity is important [38, 40, 53, 63]. Treatment started 1 h prior to the last immunization with collagen type II (CII). The prophylactic study design enabled comparison of naïve mice (healthy, non-CIA, non-treated), vehicle-treated mice (DMSO) and CIA mice treated daily with AVX001 and AVX002 (10 and 30 mg/kg, intraperitoneal) and MTX (0.3 mg/kg daily) as anti-rheumatic drug control [32, 33]. Some mice in each treatment group were killed midway through the study to enable early histopathology observations. CIA developed rapidly in mice immunized with CII; 100% incidence of CIA was observed by day 29 in CII-immunized mice, with maximum AI of 8.55 observed at 41 days post immunization when the study was terminated. The AI and incidence of all groups increased in a time-dependent mode from day 25 to 41, and AI was not significantly different between the groups in the main study and the smaller groups of mice that were killed for histology analysis at day 33, following 13 days of treatment (*p* > 0.05).

**Fig. 2:**
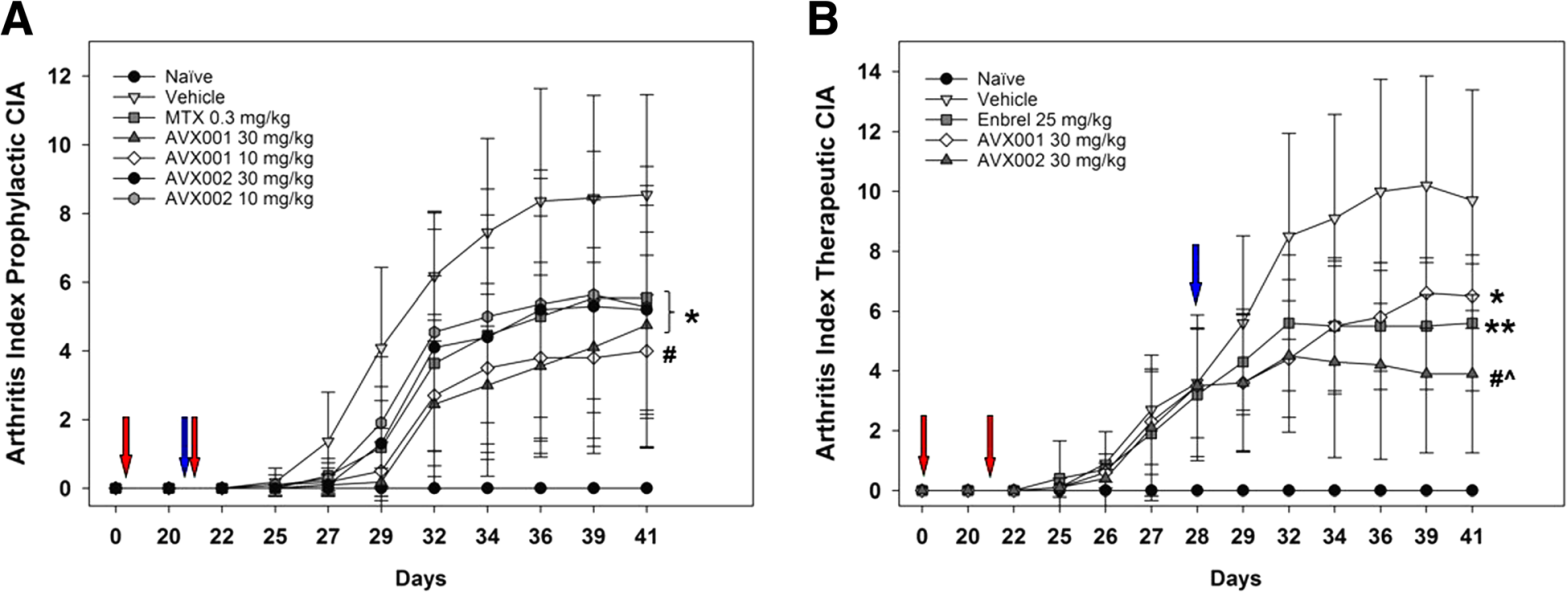
Effect of the cytosolic phospholipase A2 protein (cPLA2α) inhibitors AVX001 and AVX002 on arthritis index (AI). **a** AVX001 and AVX002 delay and reduce arthritis progression as efficiently as methotrexate (MTX) in a prophylactic collagen-induced arthritis (CIA) model (*n* = 11). **b** AVX001 and AVX002 reduce the AI in a manner comparable to Enbrel in a therapeutic CIA model (*n* = 10). *p < 0.05, **p < 0.01, ^#^*p* < 0.005 vs. vehicle, ^^^*p* < 0.01 vs. AVX001 at study termination. Red arrows mark collagen II challenge; blue arrows mark start of treatment

All treatment groups significantly reduced the AI from day 27 compared to the DMSO-treated vehicle mice (*p* < 0.05); at this early stage the effect was most prominent for AVX001 (30 mg/kg) (*p* < 0.01). The reduction in AI persisted throughout the study in all treatment groups. The effect was more evident with AVX001 (*p* < 0.005) than with MTX and AVX002 (*p* < 0.05) (Fig. 2a). Taken together, the cPLA2α inhibitors AVX001 and AVX002 delayed and reduced arthritis progression and hence exert prophylactic anti-arthritic effects that exceed or are comparable to that of the widely-used disease-modifying anti-rheumatic drug MTX.

### AVX001 and AVX002 efficiently reduce arthritis in a therapeutic CIA model

Having shown that AVX001 and AVX002 delays and ameliorate arthritis symptoms in the prophylactic CIA model and are well-tolerated in vivo, we next wanted to explore the effects of AVX001 and AVX002 when administered therapeutically, starting treatment 7 days after the last immunization. Here, Enbrel (25 mg/kg) was included for anti-rheumatic drug control [32, 33] and compared to AVX001 and AVX002 (30 mg/kg). Again, CIA developed rapidly in mice immunized with CII, with maximum AI of 10.2 observed at 39 days post immunization.

After 4 days of treatment, at day 32, all treatment groups significantly reduced the AI compared to the DMSO-treated vehicle mice (*p* < 0.05) and the reduction in AI persisted throughout the study in all treatment groups (Fig. 2b). The inhibitory effect of AVX002 exceeded that of AVX001 (*p* < 0.01) at study termination, whereas there were no significant differences between Enbrel and AVX001 or AVX002 (*p* > 0.05). Hence, the therapeutic CIA study approach confirmed the prophylactic study and show AVX001 and AVX002 exert anti-inflammatory therapeutic effects comparable to those of the TNF-blocker Enbrel.

### AVX001 and AVX002 are disease-modifying agents that reduce joint damage

**Fig. 3:**
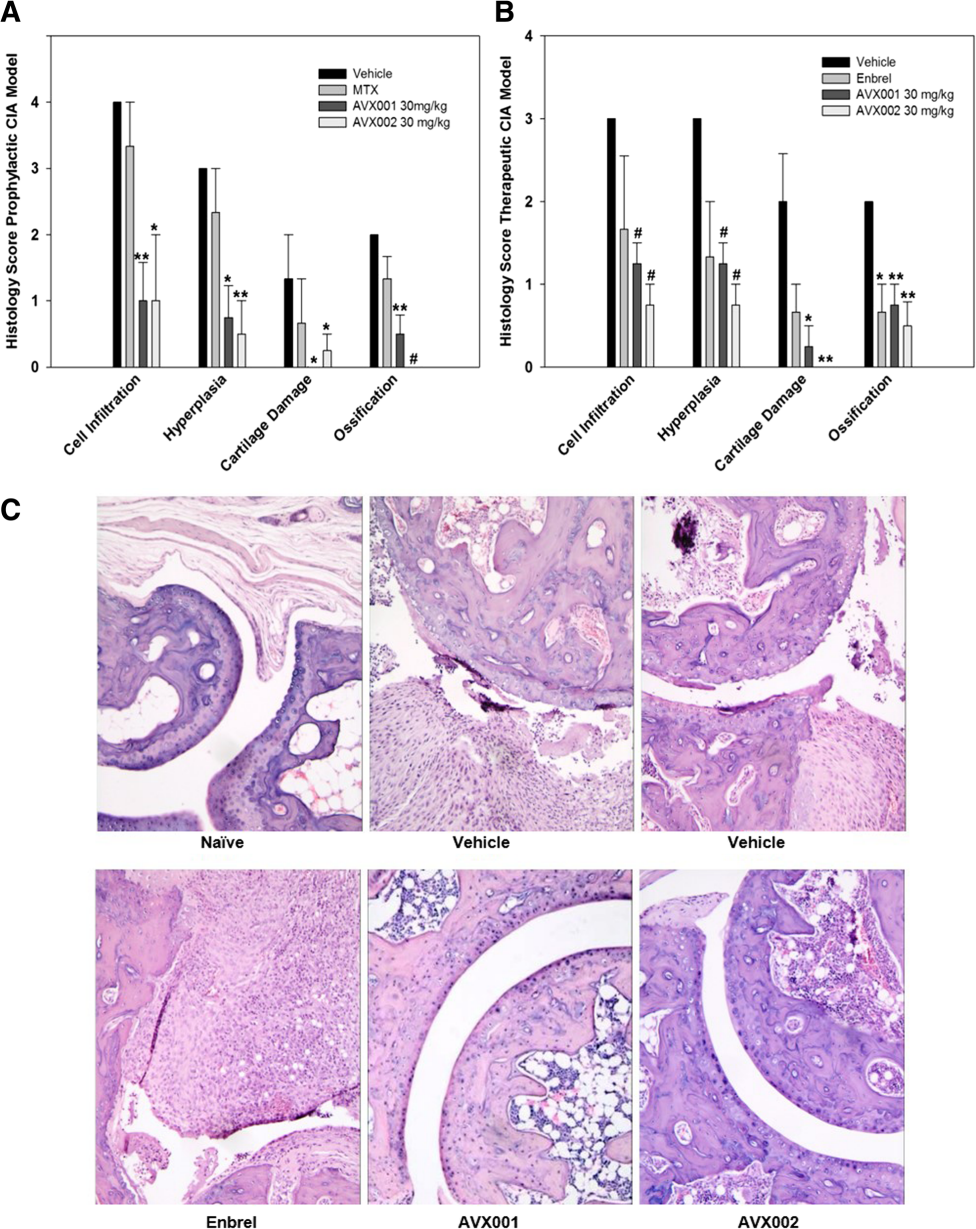
Effects of Cytosolic phospholipase A2 protein (cPLA2α) inhibitors on histopathology analysis. **a** AVX001 and AVX002 reduce arthritis and joint damage more efficiently than methotrexate (MTX) in the prophylactic collagen-induced arthritis (CIA) model. Histopathology analysis was performed on hind paws from mice killed at day 33. **b** AVX001 and AVX002 reduce arthritis and joint damage more efficiently than Enbrel in the therapeutic CIA model. Histopathology analysis was performed on hind paws from mice killed at day 41. *p < 0.05, **p < 0.01, ^#^*p* < 0.005 vs. vehicle; error bars denote standard error of mean (*n* = 3–4). **c** Representative histology analysis depicting overall joint structure in naïve, vehicle-treated and treated mice at study termination, day 41 (therapeutic study) (HE stain of paraffin sections, × 100). PGE2, prostaglandin E2

To gain insight into in situ pathological processes, joint tissue was histopathologically evaluated midway through and at termination of the prophylactic study. At day 33, having received treatment for 13 days, four mice in each group were killed and one hind paw was collected for histopathology analysis. Similarly, four mice from each treatment group and three mice from the control groups were selected at random for histopathology analysis at day 41. At day 33, with an average AI ~ 6 in the vehicle-treated mice, AVX001 and AVX002 both significantly reduced several parameters on arthritis and joint damage (*p* < 0.05 to *p* < 0.005) whereas MTX did not (*p* > 0.05) affect articular cavity and intraperitoneal tissue inflammatory cell infiltration; capillary and synovial hyperplasia; articular cartilage surface damage; and periostal and endochondral ossification. Interestingly, the ameliorating effects of the AVX compounds were more evident than that of MTX (*p* > 0.05)(Fig. 3a). Some reduction, albeit not significant, of the same arthritis and joint damage parameters were also found at the end of the main study (*p* > 0.05, results not shown).

At the end of the therapeutic study, one hind paw from four mice from each treated group and three mice from the control groups were collected for histopathology analysis. Compared with the vehicle group, AVX001 and AVX002 (30 mg/kg) both significantly reduced all four parameters of arthritis and joint damage (*p* < 0.01 to *p* < 0.005), whereas a significant effect of Enbrel (25 mg/kg) was only seen in reducing endochondral and periosteal ossification (*p* < 0.05) (Fig. 3b and c). In summary, the histological findings suggest that the cPLA2α inhibitors AVX001 and AVX002 have disease-modifying properties.

### AVX001 and AVX002 efficiently reduce plasma PGE2 levels

PGE2 is recognized as an important mediator of joint inflammation in CIA [64]. In cellular and in vivo studies, we have previously shown that cPLA2α inhibitors efficiently reduce PGE2 but still allow basal production of PGE2 [11, 12, 50]. Hence, we wanted to investigate if plasma PGE2 levels would change in response to treatment with AVX001 and AVX002 and to compare their effects with those of Enbrel and MTX.

**Fig. 4:**
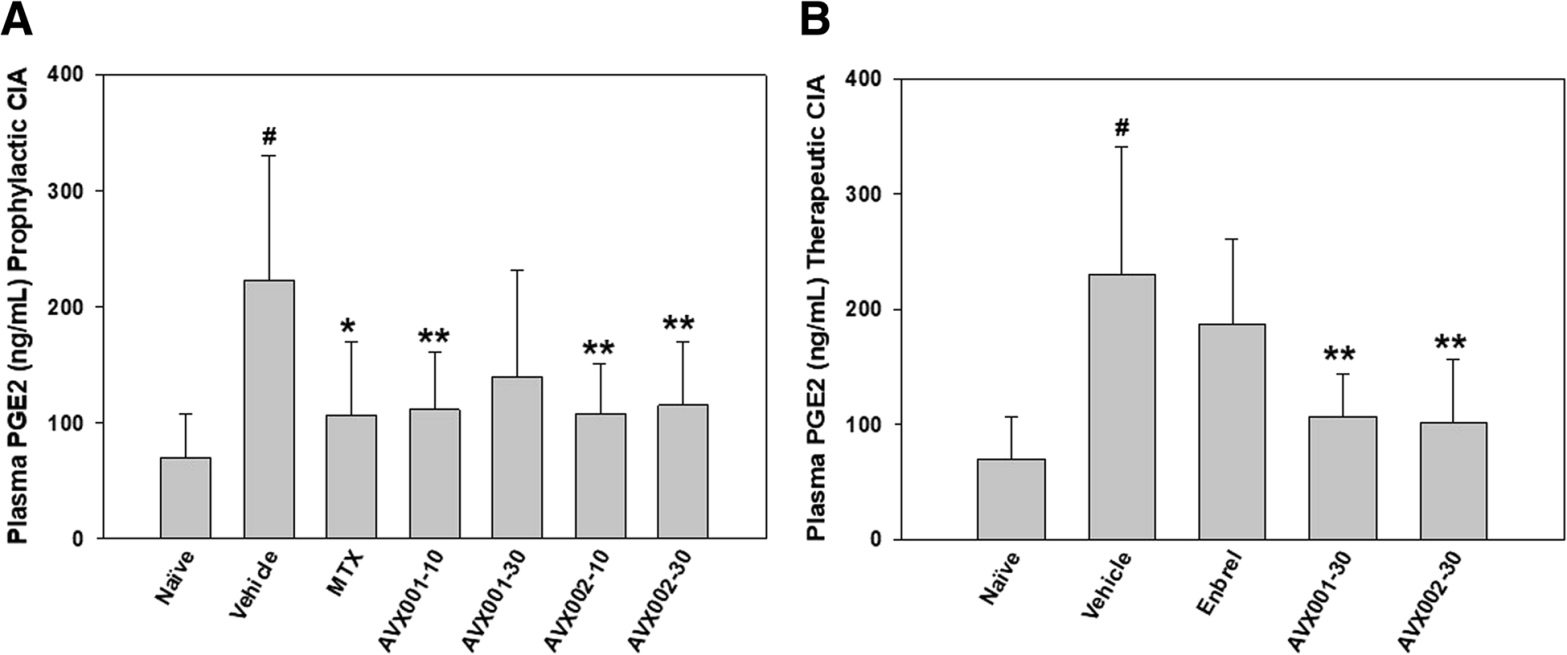
Effect of treatment on plasma prostaglandin E2 (PGE2). **a** In the prophylactic collagen-induced arthritis (CIA) study, AVX001 (10 mg/kg body weight (BW)) and AVX002 (10 and 30 mg/kg BW) significantly reduce plasma PGE2, comparable to the effect of methotrexate (MTX) (0.3 mg/kg); error bars denote standard deviation (*n* = 11). B) In the therapeutic CIA study, AVX001 and AVX002 (30 mg/kg BW) also significantly reduce plasma PGE2, whereas Enbrel (25 mg/kg BW) did not significantly reduce PGE2 (*n* = 10). *p < 0.05 and **p < 0.005 vs. vehicle, ^#^*p* < 0.001 vs. naïve

In the prophylactic study (*n* = 11), PGE2 in the DMSO-treated vehicle group was significantly elevated threefold (*p* < 0.001) compared to the non-arthritic healthy mice (Fig. 4a). The elevated PGE2 levels were reduced with all treatments; 0.3 mg/kg MTX (52% reduction, *p* < 0.05); 10 mg/kg AVX002 (51% reduction, *p* < 0.005); 10 mg/kg AVX001 (50% reduction, *p* < 0.005); 30 mg/kg AVX002 (49% reduction, *p* < 0.005); 30 mg/kg AVX001 (37% inhibition, non-significant *p* > 0.05). There were no significant differences between the treatment groups (*p* > 0.05).

Similar results were obtained In the therapeutic study (*n* = 10); PGE2 levels in the DMSO-treated vehicle group were significantly elevated threefold (*p* < 0.001) compared to the non-arthritic healthy mice (Fig. 4b). The elevated PGE2 levels were significantly reduced with both AVX treatments, but not with Enbrel; 30 mg/kg AVX002 (56% reduction, *p* < 0.005); 30 mg/kg AVX001 (54% reduction, *p* < 0.005); 25 mg/kg Enbrel (19% reduction, non-significant, *p* > 0.05). There were no significant differences between the treatment groups (*p* > 0.05).

In summary, the AVX inhibitors reduced plasma PGE2 levels by ~ 50% in both prophylactic and therapeutic modes of the CIA models; comparable to the reference drug MTX in the prophylactic study and superior to that of Enbrel in the therapeutic study.

## Discussion

In this study, our main objective was to examine the effects of the cPLA2α inhibitors AVX001 and AVX002 in vivo in the collagen induced arthritis (CIA) mice model. In the prophylactic model, both AVX inhibitors exerted an anti-inflammatory effect comparable to the reference drug methotrexate, while in the therapeutic model, the effects of AVX001 and AVX002 were comparable to those of the reference drug Enbrel. In both the prophylactic and the therapeutic models, both AVX inhibitors significantly reduced PGE2 plasma levels by ~ 50%. Hence, this study confirms that inhibition and normalization of cPLA2α activity ameliorates arthritis as evaluated clinically and histopathologically and by normalization of plasma PGE2 levels in murine models of collagen-induced arthritis. The cPLA2α inhibitors AVX001 and AVX002 perform as well as or superior to the commonly used anti-rheumatic drugs methotrexate and Enbrel in prophylactic and therapeutic modes of treatment, respectively. This indicates that cPLA2α inhibitors may halt both onset and progression of disease, and may even exert disease-modifying effects in vivo. Clearly, cPLA2α is a relevant and reachable target for small molecule drugs in RA and possibly other chronic diseases.

We have previously reported similar effects of compound Inhibitor 28 in prophylactic and therapeutic CIA study modes in mice [50]. Compound Inhibitor 28 was administered every day, whereas AVX001 and AVX002 in the current study were administered every second day following daily administration for the first 4 days. Overall, the efficacy of all three inhibitors is comparable in most parameters analyzed; reduction in arthritis index, reduction in plasma PGE2 and with regard to histopathologiy results. In the in vitro PLA2 activity assays, AVX001 and AVX002 are comparable to compound Inhibitor 28 [50]; the inhibitors are cPLA2α-specific without inhibiting iPLA2 or sPLA2 activity. In the synoviocyte model system, AA release is also inhibited by AVX001 and AVX002 in a similar manner as previously reported for compound Inhibitor 28; all displaying cellular IC50s in the ~ 0.6–1 μM range [50]. Furthermore, we have also found that AVX001 and AVX002 do not affect OA release in synoviocytes [13]. Overall, the finding that two different equipotent chemical classes of cPLA2α inhibitors - DHA- derived AVX001 and AVX002 and the compound Inhibitor 28 oxothiazole - both exert similar effects in cellular and in the in vivo model systems is a very strong indication of potency and specificity of the inhibitors.

The study shows that the AVX inhibitors reduced plasma PGE2 by ~ 50% in both prophylactic and therapeutic modes of the CIA models; comparable to the reference drug MTX in the prophylactic study and superior to that of Enbrel in the therapeutic study. When seen in conjunction with the cellular PGE2 data, these results indicate that PGE2 is a relevant biomarker for inflammation, including arthritis, and may indicate response to treatment and disease activity. The benefit of reducing PGE2 is well-known and NSAIDs are frequently used despite the associated adverse risks events [22–26].

As cPLA2α inhibitors act upstream from medications such as NSAIDs in the production of eicosanoids, but downstream compared to many biologics and immunomodulators, the cPLA2α enzyme represents an attractive target point for intervention in inflammatory chronic diseases. Theoretically, lowering the levels of the key substrate for pro-inflammatory eicosanoid production, arachidonic acid, should result in a balanced reduction of these eicosanoids. cPLA2α inhibition is well-recognized as an attractive intervention point in the inflammatory cascade for treating chronic inflammatory conditions. Furthermore, we here show that the cPLA2α-specific inhibitors AVX001 and AVX002 act to modulate - not block - inflammatory PGE2 production in vivo and in a cellular model. This property may help maintain essential host response mechanisms and housekeeping functions and thereby cause less adverse effects. In addition, normalizing cPLA2α activity should theoretically result in a balanced reduction of AA-derived metabolites as it reduces substrate availability rather than metabolizing enzyme activity, i.e. COX, LO and CYP enzyme activity. This phenomenon was not investigated in the present study, but should be elucidated in future cellular, in vivo and ex vivo model systems.

As expected, TNF blockade by Enbrel was effective in reducing the severity of arthritis in the therapeutic CIA study [65]. Like Enbrel, MTX also efficiently reduced the progression and severity of arthritis, presumably by reducing the levels of pro-inflammatory mediators such as IL-1, IL-6, TNF and prostaglandins [66–69]. Importantly, some of the pro-inflammatory effects of these cytokines may in part depend on cPLA2α activity and may even activate cPLA2α in an autocrine inflammatory loop. Interestingly, the histological findings indicate that cPLA2α inhibitors such as AVX001 and AVX002 have disease-modifying properties that are superior to those of MTX and Enbrel. There are several reports that the TNF blockade is less efficient than the IL-1β blockade when it comes to joint destruction, even if overall clinical and/or serological findings are convincing. TNF may be more involved in inflammation whereas IL-1β may also be a potent regulator of tissue destruction [70–72]. Also, there are reports that MTX is better than TNF blockers with respect to several histopathological parameters and MTX is indeed reported to inhibit PGE2 both in cellular models and in animal models [66, 68, 73]. Overall, this fit well with our findings.

**Fig. 5:**
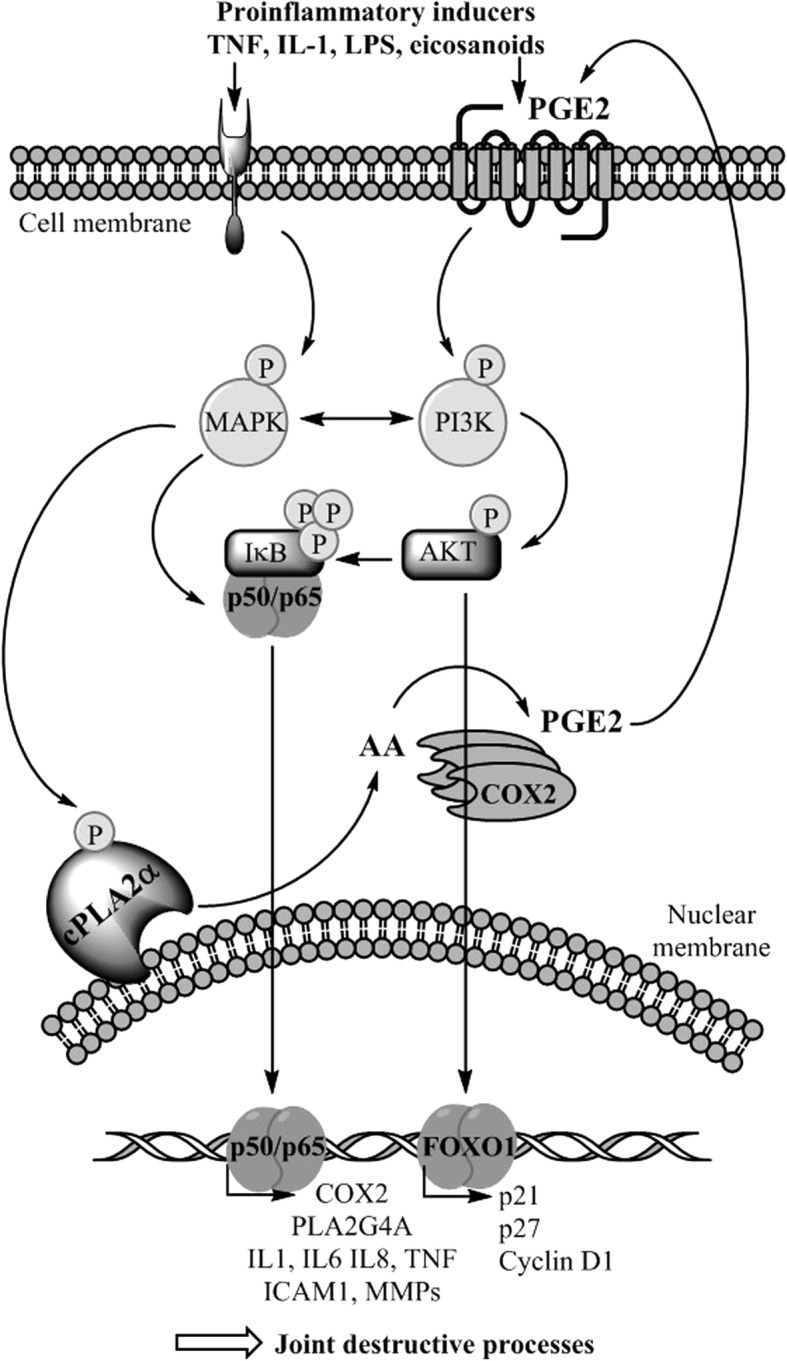
Hypothesized molecular understanding of the disease-modifying anti-rheumatic drug (DMARD) properties of cytosolic phospholipase A2 protein (cPLA2α) inhibitors in the inflamed joint. Different types of cells are activated by the ongoing inflammatory process; chondrocytes, osteoblasts, osteoclasts, synoviocytes, immune cells and endothelial cells. Cell activation involves increased activity of many signaling pathways that involve cPLA2α. Upon reduced cPLA2α activity, reduced arachidonic acid (AA availability) and eicosanoid levels may lead to reduced activity of transcription factors such as NF-κB and FOXO1, that regulate the transcription and synthesis many mediators of inflammation and proliferation. Reduced levels of pro-inflammatory mediators and altered levels of cell cycle regulators may explain how normalization of cPLA2α activity may help reduce the ongoing joint destructive processes. LPS, lipopolysaccharide; MAPK, mitogen-activated protein kinase; PGE2, prostaglandin E2; COX2, cyclooxygenase 2

Several cellular and molecular mechanisms may help explain the observed DMARD properties of the cPLA2α inhibitors, hypothesized in Fig. 5. For instance, we have previously shown that cPLA2α inhibitors yield reduced PGE2 due to reduced AA availability, reduced COX2 (PTGS2) and cPLA2α (PLA2G4A) gene transcription, all of which are anti-inflammatory effects [12]. Reduced levels of PGE2 and other eicosanoids in the inflamed joint may in turn imply less activation of MAPK signaling pathways [74], and ultimately less activation of transcription factors such as NF-κB [8, 9, 11, 62]; altogether calming inflammation, tissue degradation and loss of organ function. Furthermore, we have previously reported that cPLA2α may be subject to auto-regulation as inhibition of cPLA2α activity leads to reduced expression of PLA2G4A mRNA in response to TNF and that cPLA2α regulates mediators of bone and cartilage destruction, angiogenesis and neutrophil recruitment, such as stromelysin-1 (matrix metalloproteinase 3 (MMP3)), IL-6 and IL-8, COX2 and PGE2 [75, 76]. The effects of such mediators in bone resorption has also been shown to be PGE2-dependent [39, 76].

A key event in the pathogenesis of arthritis is the influx of mononuclear cells to the synovium, the local activation of such cells and the formation of new blood vessels in the synovium. The resulting synovitis progresses into the formation of the bone and cartilage degrading pannus [4]. In the inflamed joint, neutrophils are considered important in RA as they are located at the pannus-erosion boundary and are abundant in the synovial fluid [77, 78]. cPLA2α may regulate the infiltration of immune cells by regulating the expression of adhesion molecules such as ICAM-1 [62] and chemokines such as IL8 [12]. Neutrophil extravasation is initiated by endothelial expression of ICAM-1, an adhesion molecule that is induced by pro-inflammatory cytokines, and it is shown that ICAM-1 expression is regulated by cPLA2α [62, 79]. Furthermore, direct coupling of neutrophil infiltration and cPLA2α is evidenced by Raichel et al. [39] where a cPLA2α knockdown by siRNA reduced neutrophil infiltration, tissue damage and reversed the course of disease, all in line with our results. In response to the cPLA2α inhibitors AVX001 and AVX002, the arthritis index that describes the number of inflamed joints and the degree of joint swelling and erythema, was significantly reduced in both prophylactic and therapeutic study modes. In parallel, the AVX inhibitors significantly reduced several parameters of arthritis and joint destruction and plasma levels of PGE2, the latter suggesting that the AVX inhibitors have indeed hit the cPLA2α target. These anti-inflammatory and disease-modifying effects may well be correlated to reduced levels of vasodilating and angiogenic eicosanoids due to reduced availability of the substrate arachidonic acid. Reduced joint swelling, erythema, bone and cartilage destruction may in part be due to reduced levels of PGE2 [19, 80, 81], although other eicosanoids most likely are affected as well.

Angiogenesis is another important process that controls inflammation and tissue function, and angiogenic processes are also shown to be regulated by cPLA2α and eicosanoids [82]. Recently, we showed that the cPLA2α inhibitor AVX235 profoundly affects vascularization in xenograft tumors and reduced tumor growth [51]. Although it was not investigated in this study, it is possible cPLA2α inhibition may even affect the proliferation of endothelial cells and synoviocytes in the inflamed joint, as recent studies describe that inhibitors of cPLA2α may induce growth arrest in several types of cells via FOXO1 inhibition [83, 84]. Taken together, we propose that the observed DMARD effects of AVX001 and AVX002 may be explained by their capacity to limit immune cell influx, angiogenesis and possibly also by inducing cell cycle arrest of various types of cells in the inflamed joint, thereby limiting synovial hyperplasia and pannus formation, and consequently preventing tissue degradation.

## Conclusion

This study confirms that cPLA2α is a key therapeutic target in RA [37–39, 50]. We show that reducing, not blocking, cPLA2α activity delays collagen-induced arthritis progression and severity in mice, similar or superior to MTX and Enbrel in prophylactic and therapeutic modes of treatment, respectively. In both modes of treatment, there were significant improvements in all histopathology parameters evaluated; both AVX001 and AVX002 inhibitors of cPLA2α reduced the scores for joint inflammation and joint destruction more potently than MTX and Enbrel. In conclusion, acting on a less superior target in the inflammatory cascade than DMARDs, and upstream of NSAIDs, the cPLA2α inhibitors exhibit both anti-inflammatory and disease modifying properties of the small molecule therapeutics that most likely may be associated with less adverse effects compared to NSAIDs and DMARDs [50] and may emerge as important anti-rheumatic drugs, alone or in combination with existing anti-rheumatic drugs.

## Abbreviations

AA: Arachidonic acid
AI: Arthritis index
ATK: Arachidonyl trifluorketone
BSA: Bovine serum albumin
BW: Bodyweight
CCL2/MCP-1: Monocyte chemoattractant protein-1
COX: Cyclooxygenase enzymes
cPLA2α: Cytosolic phospholipase A2 protein
Ctrl: Control
CVD: Cardiovascular disease
CYP: Cytochrome P enzymes
DHA: Docosahexaenic acid
DMARD: Disease-modifying anti-rheumatic drugs
DMSO: Dimethyl sulfoxide
DTT: Dithiothreitol
EIA: Enzyme immunoassay
FOXO1: Forkhead box protein O1
HEPES: Hydroxyethyl piperazineethanesulfonic acid
HRS: Hours
IC50: Half maximal inhibitory concentration
ICAM-1: Intercellular adhesion molecule 1
IL-1β: Interleukin-1 beta
IL-6: Interleukin-6
IL-8: Interleukin-8
LO: Lipoxygenase enzymes
MAPK: Mitogen-activated protein kinase
MMP: Matrix metalloproteinases
MTX: Methotrexate
NF-kB: Nuclear factor kappa-light-chain-enhancer of activated B cells
NSAID: Non-steroid anti-inflammatory drugs
OA: Oleic acid
OD: Optical density
PAPC: 1-palmitoyl-2-arachidonoyl-sn-glycero-3-phosphocholine
PGE2: Prostaglandin E2
PIP2: Phosphatidylinositol-4,5-bisphosphate
RA: Rheumatoid arthritis
SD: Standard deviation
siRNA: Small interfering RNA
TNF: Tumor necrosis factor
